# Translating Molecular Subtypes into Cost-Effective Radiogenomic Biomarkers for Prognosis of Colorectal Cancer

**DOI:** 10.3390/diagnostics16020273

**Published:** 2026-01-14

**Authors:** Baowen Gai, Xin Duan, Chenghang Li, Chuling Hu, Minyi Lv, Jiaxin Lei, Runxian Wang, Feng Gao, Du Cai

**Affiliations:** 1 Department of General Surgery (Colorectal Surgery), The Sixth Affiliated Hospital, Sun Yat-sen University, Guangzhou 510655, China; gaibw@mail2.sysu.edu.cn (B.G.);; 2 Guangdong Provincial Key Laboratory of Colorectal and Pelvic Floor Diseases, The Sixth Affiliated Hospital, Sun Yat-sen University, Guangzhou 510655, China; 3 Biomedical Innovation Center, The Sixth Affiliated Hospital, Sun Yat-sen University, Guangzhou 510655, China; 4 School of Biomedical Engineering, Shenzhen Campus of Sun Yat-sen University, Shenzhen 518000, China; 5 Department of Oncology, Cancer Institute, Peking University Shenzhen Hospital, Shenzhen Peking University-Hong Kong University of Science and Technology (PKU-HKUST) Medical Center, Shenzhen 518036, China; 6 School of Medicine, Shenzhen Campus of Sun Yat-Sen University, Shenzhen 518107, China; 7 Department of Gastrointestinal Surgery, The Fifth Affiliated Hospital of Sun Yat-sen University, Zhuhai 519000, China

**Keywords:** colorectal cancer, molecular subtypes, intratumor heterogeneity, prognosis, radiogenomic, biomarkers

## Abstract

**Background:** Colorectal cancer (CRC) is currently the third most common cancer worldwide, with high heterogeneity and poor prognosis. Gene expression-based molecular subtypes can effectively dissect tumor heterogeneity, but their clinical translation remains challenging. This study aims to conduct radiogenomic analysis regarding molecular subtypes and establish prognostic signatures for survival prediction of colorectal cancer. **Methods**: In this retrospective study involving 2948 CRC patients from 8 cohorts, we utilized a supervised deep learning framework to extract quantitative feature representations of molecular subtypes. Through correlation analysis, we selected key gene expression features related to these subtypes to establish a prognostic signature. A similar pipeline was applied to derive a non-invasive radiomic prognostic signature. Finally, we validated the prognostic value of both signatures in multiple cohorts and explored their biological interpretation. **Results**: We successfully established a molecular subtype-associated gene signature and a non-invasive radiogenomic signature. The gene signature classified patients into high-risk and low-risk groups with significantly different prognoses. The low-risk group had a better prognosis and showed a greater potential benefit from immunotherapy. Similarly, the radiogenomic signature exhibited characteristics related to molecular subtypes and comparable performance in prognostic prediction. Multivariate analysis confirmed the independent prognostic value of both signatures. In summary, this retrospective study demonstrates that our framework translates molecular subtypes into cost-effective biomarkers for risk stratification and treatment guidance.

## 1. Introduction

Colorectal cancer (CRC) is a prevalent gastrointestinal malignancy with the third highest incidence and the third highest mortality rate in the world [1]. Despite considerable advancements in clinical interventions for colorectal cancer, the overall 5-year survival rate for CRC remains approximately 65% [2]. Surgery remains the primary means of curative treatment [3]. However, some patients develop local recurrence and distant metastases after surgery. At the same time, patients with the same clinical or pathologic status show unpredictable clinical outcomes, even when receiving similar treatment [4]. This variability is due to the tumor heterogeneity of CRC, resulting in variable treatment response rates and patient outcomes [5]. The existing risk stratification system based on TNM staging relies solely on anatomical extent and fails to capture distinct biological behaviors, resulting in the inability to accurately individualize risk prediction and treatment [6,7]. Therefore, there is an urgent need to provide more accurate risk prediction and prognostic stratification for colorectal cancer.

The highly diverse phenotypic and molecular characteristics of the tumor lead to differences in growth rate, invasion and metastasis, drug sensitivity, prognosis, and other aspects, namely the malignancy heterogeneity [8]. Numerous clinical trials have demonstrated the high heterogeneity of CRC, highlighting the potential for improved outcomes through treatment tailored to the tumor’s molecular and histological characteristics [9,10]. To enhance the classification of CRC and better understand its tumor heterogeneity, the International Colorectal Cancer Subtyping Consortium (CRCSC) successfully identified four consensus molecular subtypes (CMS) by analyzing data from more than 4000 primary tumor samples in 18 cohorts [11]. CMS aligns with established biological variations in CRC and helps to address the issue of inter-tumor heterogeneity [12]. The various subtypes have different molecular characteristics and different clinical features. While CMS captures to some extent the biological information of tumor heterogeneity [13], its prognostic performance varies across different disease stages, and its direct utility in guiding clinical decisions remains limited [14]. Additionally, the application of CMS requires high-throughput sequencing, which imposes high medical costs and presents a major barrier to its widespread clinical application of CMS.

Radiogenomics, as an interdisciplinary field, aims to investigate the intricate relationship between radiomic features derived from medical imaging and gene expression profiles, with the primary objective of improving disease prognosis and the accuracy of predicting treatment response. Several studies have identified the potential of radiogenomics in molecular subtyping, disease diagnosis and treatment prediction [15,16,17,18,19]. Zeng et al. developed a radiogenomic signature to predict mutations and molecular subtypes in renal clear cell carcinoma [20]. Smedley et al. utilized deep neural networks to infer the association of radiogenomic signatures for more accurate diagnosis of non-small cell lung cancer [21]. However, in colorectal cancer, most existing radiogenomic studies primarily focus on predicting single gene mutations or general survival, failing to capture the comprehensive biological information embedded in CMS.

Here, using the tumor heterogeneity information embedded in molecular subtypes, we developed a translational framework to acquire low-throughput gene expression or radiogenomic signatures associated with cancer patients’ prognosis. This approach has the potential to not only reduce healthcare costs for patients but also assist clinicians in making diagnostic and therapeutic decisions for patients with CRC.

## 2. Materials and Methods

### 2.1. Data Collection and Preprocessing

A total of 6 public cohorts were collected in this study, including TCGA, GSE39582, GSE14333, GSE17538, GSE37892, and GSE33113. TCGA (*n* = 624) was obtained from the Firehose Broad GDAC portal. All samples retained gene expression data on the Hi-Seq platform only. Furthermore, we obtained the corresponding CMS classification labels for the samples from the CRCSC Synapse repository. GSE39582 (*n* = 566) were obtained from the sequencing cohort from the French CIT Project [22]. Meta-Validation (*n* = 742) was merged from four datasets including GSE14333 (*n* = 290), GSE17538 (*n* = 232), GSE37892 (*n* = 130) and GSE33113 (*n* = 90). We manually obtained the data for the microarray datasets from the Gene Expression Omnibus (GEO) database. After downloading the datasets, the probeset IDs were linked to gene symbols through the utilization of the corresponding gene annotations (GPL570). In cases where multiple probesets were mapped to the same gene symbol, only the ones with the maximum average expression level were kept to capture the most robust biological signal [23]. To address non-biological technical biases and ensure comparability, we applied the ComBat algorithm from the ‘sva’ R package (version 3.58.0, Bioconductor) [24] to remove batch effects prior to downstream analysis. In all collected public cohorts, we excluded all samples with incomplete survival information.

To further assess the robustness of our gene signature, three additional independent cohorts were included for validation: GSE38832 (*n* = 122), GSE31595 (*n* = 37), and GSE75316 (*n* = 39). Gene expression data for these cohorts were downloaded from GEO and processed using a similar pipeline to the other datasets.

### 2.2. In-House RNA-Seq and CT Cohort

The COCC (Clinical Genomic Study of Colorectal Cancer in China) cohort is the colorectal subproject of the ICGC-ARGO (International Cancer Genome Consortium to Accelerate Research in Genomic Oncology, https://www.icgc-argo.org/ accessed on 10 December 2024). This project is led by The Sixth Affiliated Hospital of Sun Yat-sen University and plans to conduct multiomic sequencing for Chinese colorectal cancer patients, and currently, RNA-seq data from 587 samples were included in this study. For the treatment of samples, fresh frozen tissues were subjected to TRAzol-based extraction to isolate the total RNA. To eliminate rRNA, the MGIEasy rRNA Depletion Kit (MGI Tech Co., Ltd., Shenzhen, China, 32 RXN, 1000005953) was employed following the manufacturer’s guidelines. For the generation of sequencing libraries, the conventional random primer method was employed thereafter. Utilizing the DNBSEQ-T1 platform (BGI), whole transcriptome sequencing was conducted. Prior to alignment, raw sequencing data underwent rigorous quality control. Adaptor sequences and low-quality reads were filtered out to generate clean reads, ensuring that only high-quality data (Q30 > 85%) were used for downstream analysis. Alignment of the reads to the GRCh38/hg38 reference genome was accomplished through the utilization of HISAT [25]. Subsequently, transcript expression quantification was performed using RSEM [26].

The study also included a total of 768 patients with preoperative CT data from The Sixth Affiliated Hospital of Sun Yat-sen University. Among them, those with matched RNA-seq data were used as the training cohort (*n* = 233). The rest served as the validation cohort (*n* = 535). In our study, all enhanced CT scans were acquired in Digital Imaging and Communication in Medicine (DICOM) format. Two experienced radiologists from the Sixth Affiliated Hospital of Sun Yat-sen University used ITK-snap to manually delineate the tumor region of interest and generate a 3D segmentation, and the results were cross-validated by each other to ensure reliability. Subsequently, the processing of the images and the extraction of the features were performed by using the ‘Pyradiomics’ package (version 3.1.0) [27] on the Python (version 3.8) platform. Specifically, standardized preprocessing steps, including resampling to uniform voxel spacing and Z-score normalization, were applied within the pipeline to ensure data consistency and feature robustness.

### 2.3. Study Design

**Figure 1:**
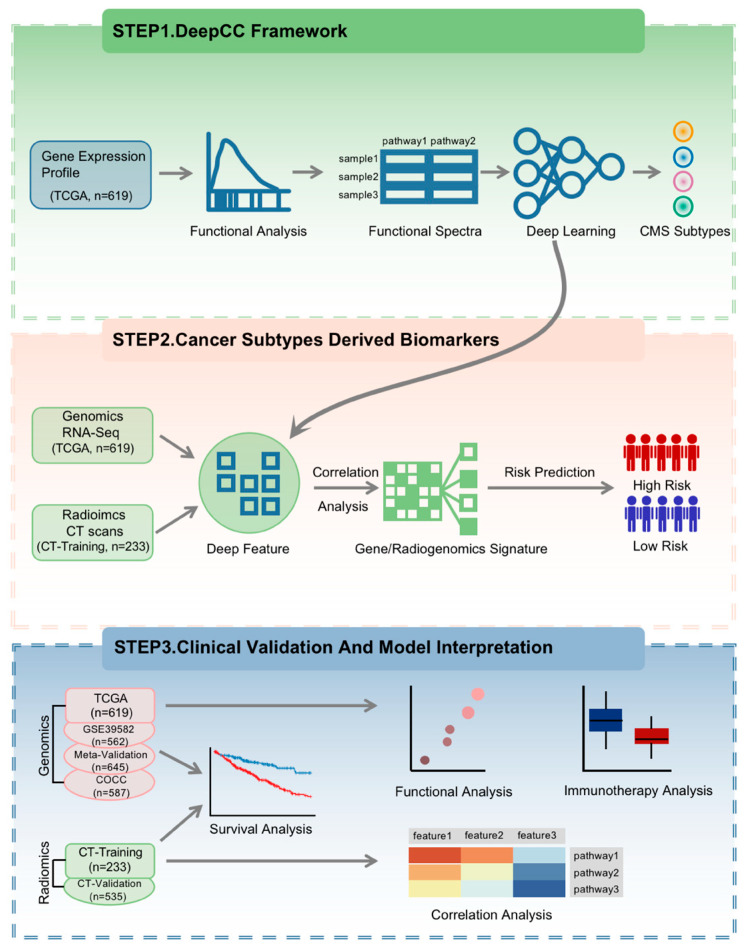
Study design. The workflow of our study. Firstly, feature representations of CMS were extracted by a supervised deep learning framework. Secondly, the mapping relationship between genomic/radiogenomic features and deep features was used to construct prognostic signatures. Finally, we validated the prognostic value of gene/radiogenomic signatures and explored their biological interpretation. Different background colors represent distinct stages of the study workflow: the green panel (top) indicates Step 1 (DeepCC Framework); the orange panel (middle) indicates Step 2 (Cancer Subtypes Derived Biomarkers); and the blue panel (bottom) indicates Step 3 (Clinical Validation and Model Interpretation). Additionally, in the risk prediction section, red and blue icons represent high-risk and low-risk groups, respectively.

This study consists of three main steps (Figure 1). (1) This step was based on our previously published DeepCC framework, utilizing the exact same parameter settings as the original study to ensure reproducibility [28]. Briefly, we first converted high-throughput RNA-seq data from tumor samples into functional spectra using gene set enrichment analysis (GSEA) [29]. These functional spectra were then used as input for the established DeepCC classifier, a fully connected multi-layer neural network designed to classify CMSs. For this study, instead of using the final classification output, we extracted the feature vector from the last hidden layer of the DeepCC model. These vectors, referred to as CMS-associated deep features, represent a learned, low-dimensional embedding of the complex biological information related to CMSs. (2) CMS-associated biomarkers identification. In order to develop a clinically available assay based on deep features, we initially calculated the top 10 genes or radiomic features (positive or negative) associated with each deep feature. We then selected the top three variable genes or radiomic features (as evaluated by median absolute deviation, MAD) for each deep feature to obtain CMS-associated signatures. The CMS-associated signature was used to predict the prognosis of CRC patients. (3) Clinical validation of CMS-associated biomarkers. To demonstrate the prognostic differences between patients predicted into high-risk and low-risk groups, survival analysis was utilized. Additionally, functional analysis was conducted to provide a biological rationale for CMS-associated signatures and offer guidance for immunotherapy.

### 2.4. Functional Analysis

To explore the biological characteristics of the identified high-risk group and low-risk group, we used the Bioconductor package ‘DESeq2′ (version 1.50.2) [30] for differential gene analysis using Benjamini–Hochberg adjustment. Subsequently, pathway enrichment analysis for GO and KEGG was performed using gProfiler, utilizing all annotated Homo sapiens genes as the background universe. Significant pathways were identified based on a Benjamini–Hochberg adjusted *p* < 0.05. Further gene set enrichment analysis (GSEA) for important pathways was performed using the R ‘HTSanalyzeR2’ package. By leveraging information from a previous publication [31], we acquired feature gene panels specific to each type of immune cell. Subsequently, we utilized single-sample gene set enrichment analysis (ssGSEA) [32] to quantify the relative infiltration of 28 immune cell types within the tumor microenvironment.

### 2.5. Immunotherapy Analysis

To evaluate the sensitivity of immunotherapy, we calculated several immunotherapy indicators in CRC patients. TIDE score [33] is considered to be a predictor of response to immunotherapy. For the calculation of the TIDE score, we use the ‘tidepy’ package [33] in Python. Immunophenoscore (IPS) [31] refers to the four main components that determine immunogenicity. IPS (range 0–10) was calculated based on gene expression in representative cell types. We calculated IPS scores using the ‘IOBR’ package [34] in R. MIRACLE scores [35] were calculated using the ‘MIRACLE’ package in R. TMB was calculated by the ‘maftools’ package in R. A related study indicated that the expression levels of key genes associated with immune checkpoints may correlate with the clinical outcome of immunotherapy [36]. In addition, we calculated the expression of 9 immune checkpoint targets: BTLA, PD-L1, CD276, CTLA-4, TIM-3, LAG3, PD-1, PD-L2, and TIGIT in different risk groups. In addition, we obtained three immunotherapy cohorts from the Tumor Immunotherapy Gene Expression Resource (TIGER) [37] for validation.

### 2.6. Statistics

We used R software (version 4.1.2) for statistical analysis. For prognostic signature development, feature selection and model construction were performed exclusively within the training cohort. Features were first screened using a univariate Cox proportional hazards regression model. Significant features were then incorporated into a multivariate Cox model to build the final signature and calculate a risk score (RS) for each patient. The RS was calculated as the linear combination of the expression levels of signature features, weighted by their multivariate Cox regression coefficients. The optimal cutoff for the RS was determined in the training cohort using time-dependent ROC curve analysis, selecting the point with the maximum Youden index. Survival curves were plotted using the Kaplan–Meier method and compared with the log-rank test. To assess if the RS was an independent prognostic factor, it was included in a multivariate Cox analysis with other clinical variables. The predictive performance of the model was evaluated by the concordance index (C-index). For comparisons between the high- and low-risk groups, the Wilcoxon rank-sum test was used for continuous variables and the chi-square or Fisher’s exact test for categorical variables. Pearson correlation was used to measure the association between continuous variables. Statistical significance was considered at a two-sided *p* < 0.05.

## 3. Results

### 3.1. Construction of the CMS-Associated Gene Signature

**Figure 2:**
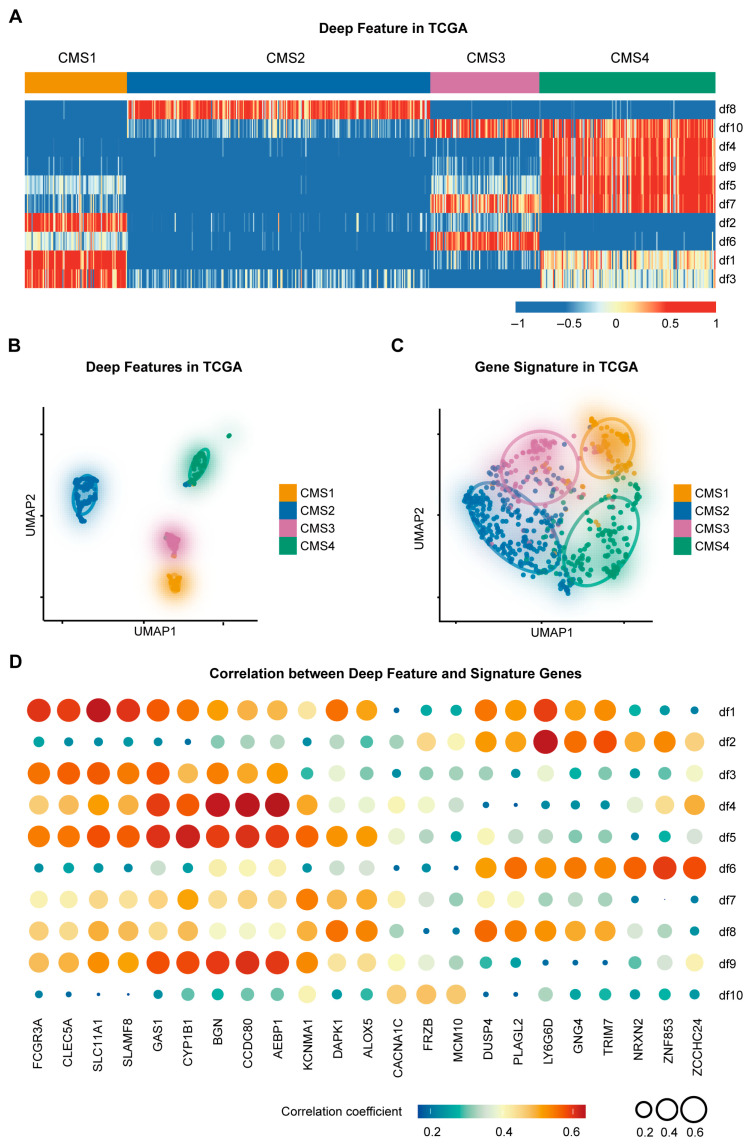
Deep learning-derived features successfully capture CMS-specific biological information and enable the construction of a robust gene signature. (**A**) The correlation heatmap of ten deep features and four CMSs. Colors represent correlation coefficients. Differences in deep feature expression among subtypes were statistically significant (Kruskal–Wallis test, *p* < 0.0001). (**B**) Uniform Manifold Approximation and Projection (UMAP) visualization of ten deep features showing subtype separation. Statistical analysis of UMAP coordinates confirmed significant separation among CMS subtypes (Kruskal–Wallis test, *p* < 0.0001). (**C**) UMAP visualization of 24 signature genes (*p* < 0.0001, Kruskal–Wallis test). (**D**) Correlation plot illustrating the relationship between deep features and signature genes. The color and size of the circles represent the correlation coefficient.

This study included a total of 2948 CRC patients from six public RNA-seq cohorts, one in-house RNA-seq cohort, and one in-house CT cohort. The clinical characteristics of all patients are presented in Table 1. The CMS-associated gene signature was developed based on the discovery cohort (TCGA) using deep learning methods. Specifically, we extracted the last hidden layer of a CMS-supervised classifier as CMS-associated deep features and constructed the gene signature by selecting the genes highly correlated with deep features and prognosis (Table S1). The heatmap showed a strong correlation between the four CMSs and the deep features (Kruskal–Wallis test, *p* < 0.0001; Figure 2A). Uniform manifold approximation and projection (UMAP) method revealed excellent separation between the different subtypes of CMS by deep features (Figure 2B). These results confirmed that CMS-supervised deep features have biological information that is strongly correlated with CMS. We then explored the characteristics of the CMS-associated gene signature. The results from UMAP analysis also showed good separation of the four CMS subtypes for the signature genes (Figure 2C). Through correlation analysis, we identified representative genes driving these features. SLC11A1 and FCGR3A were highly correlated with deep feature 1, LY6G6D was highly correlated with deep feature 2, and BGN, CCDC80, and AEBP1 were highly correlated with deep feature 4 (Figure 2D). Collectively, these analyses demonstrate that our deep learning-derived gene signature successfully captures the intrinsic biological heterogeneity of CMS subtypes, translating complex features into interpretable molecular markers.

### 3.2. Prognosis Assessment of CMS-Associated Gene Signature

**Figure 3:**
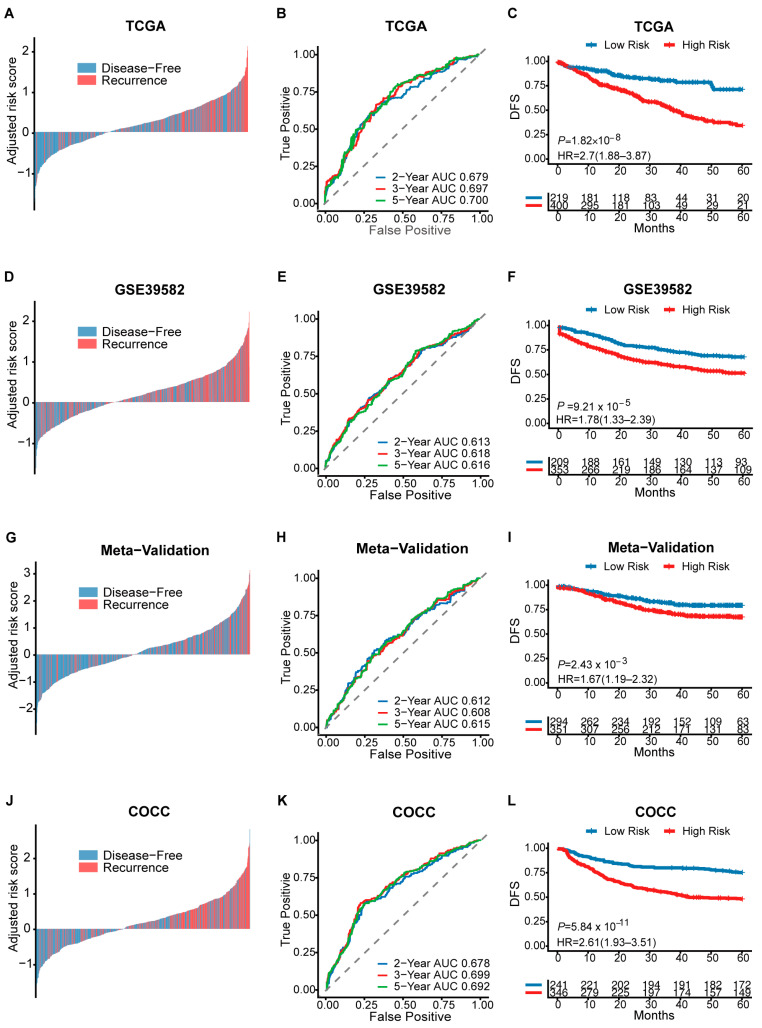
The CMS-associated gene signature provides robust prognostic stratification. (**A**,**D**,**G**,**J**) Waterfall plots show the relationship between gene signature risk scores and recurrence status. (**B**,**E**,**H**,**K**) Time-dependent ROC curves show the ability of the gene signature to discriminate between 2-year, 3-year, and 5-year recurrence status. The grey dashed line represents the reference line of no discrimination (AUC = 0.5). (**C**,**F**,**I**,**L**) Kaplan–Meier curves reveal significant associations between gene signature and disease-free survival (DFS) in four cohorts.

To assess the prognostic value of our CMS-associated gene signature, we used TCGA (*n* = 624) as a training cohort and derived a risk score (RS) from a Cox proportional risk model. The optimal RS cutoff for classifying CRC patients into high-risk or low-risk groups was determined to be −0.14. Providing the primary evidence for prognostic stratification, Kaplan–Meier analysis demonstrated that the high-risk group had a significantly lower disease-free survival rate compared to the low-risk group (DFS, log-rank *p* < 0.0001, HR [95%CI]: 2.59 [1.84–3.64], Figure 3C). Complementing this primary finding, time-dependent ROC curves and waterfall plots confirmed the model’s high predictive accuracy and distinct risk separation. The model’s robustness was further assessed by validating it in two external cohorts, GSE39582 (*n* = 562) and Meta-Validation (*n* = 645), as well as our in-house cohort, COCC (*n* = 587). The gene signature demonstrated reliable time-dependent AUC values (ranging from ~0.61 to 0.70), which effectively captured the inherent heterogeneity of CRC prognosis. Specifically, patients in the high-risk group had significantly worse prognostic outcomes across multiple cohorts, including GSE39582 (HR = 1.8, *p* < 0.0001), Meta-Validation (HR = 1.63, *p* < 0.01), and COCC (HR = 2.6, *p* < 0.0001) (Figure 3), confirming the model’s robustness across different populations. Univariate and multivariate analyses showed that the RS could be used as an independent factor for prognostic prediction (Table S2).

To further confirm the robustness of our signature, we performed survival analysis on each of the four individual datasets that comprise the Meta-Validation cohort, as well as in three additional independent cohorts. The signature consistently and effectively stratified patients into high- and low-risk groups in the majority of these datasets. In two cohorts, the stratification did not reach statistical significance, potentially due to limited sample size or cohort-specific characteristics; however, a trend of separation between the survival curves of the high- and low-risk groups was still observable (Figures S1 and S2).

To provide biological transparency to the risk stratification, we employed SHapley Additive exPlanations (SHAP) analysis to interpret the prognostic model. This analysis did not merely rank genes but quantified the precise contribution of each signature gene to the patient’s Risk Score (Table S3). The analysis identified SLAMF8, SLC11A1, and CLEC5A as the features with the highest overall impact on predicting patient risk. Validating the biological plausibility of our signature, we compiled existing experimental evidence for the top 5 most impactful genes, confirming their established roles in colorectal cancer progression and prognosis (Table S4). Furthermore, by visualizing individual cases, the SHAP analysis illustrates how the combined expression values of the signature genes drive the model’s output towards either a high-risk or a low-risk prediction (Figure S3), confirming that our risk scoring system operates on biologically interpretable grounds.

### 3.3. High-Risk Group Was Associated with CMS4

**Figure 4:**
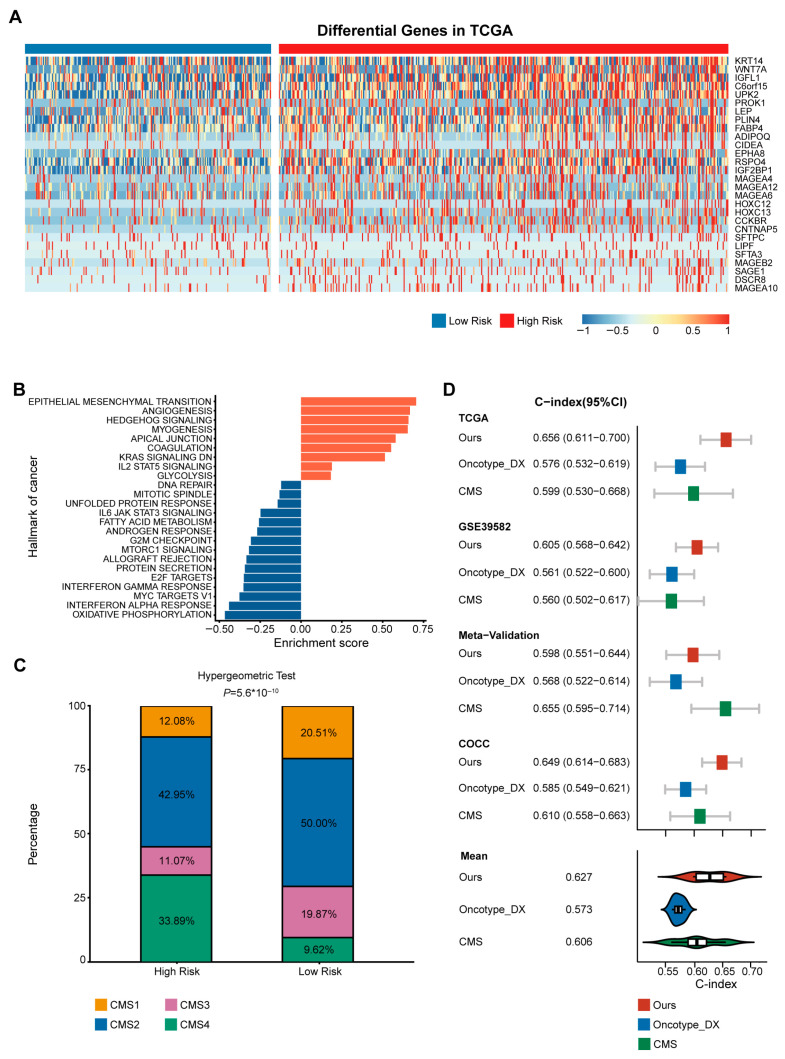
Functional analysis revealed that the high-risk group may be primarily associated with CMS4. (**A**) Heatmap illustrating the differentially expressed genes in the high and low risk groups. Displayed genes were selected based on the criteria: FDR-adjusted *p* < 0.05 and |log2 fold change| > 2. (**B**) GSEA revealed significant upregulation of the epithelial–mesenchymal transition (EMT), angiogenesis, and myogenesis pathways in the high-risk group, while the interferon alpha pathway was significantly downregulated. The orange bars represent positively enriched pathways, while the blue bars represent negatively enriched. (**C**) Barplot depicting the distribution of CMSs in the high and low risk groups. CMS4 was found to be more prevalent in the high-risk group, with statistical significance determined using a hypergeometric test. (**D**) Comparison of the C-index for prognostic prediction between our model, the Oncotype_DX model, and the CMS model.

In exploring the biological characteristics of our gene signature, we identified differentially expressed genes between the predicted high-risk and low-risk groups in the discovery cohort. Using stringent selection criteria (FDR-adjusted *p* < 0.05 and |log2 fold change| > 2), we identified 28 significantly dysregulated genes (Figure 4A). Critically, rather than functioning as isolated markers, these genes collectively indicate the activation of specific biological programs. Gene set enrichment analysis (GSEA) revealed significant up-regulation in the epithelial–mesenchymal transition (EMT), angiogenesis, and myogenesis pathways, as well as significant down-regulation in the interferon-alpha response pathway, in the high-risk group (Figure 4B). These findings are consistent with the biological profile of CMS4, a mesenchymal subtype with a poor prognosis, as reported in previous studies [11]. This suggests that our predicted poor prognosis in the high-risk population may be attributed to CMS4. We tested this hypothesis by analyzing the proportion of each CMS subtype in patients from the high-risk and low-risk groups and found a higher proportion of CMS4 patients in the high-risk group than in the low-risk group. Quantitative analysis revealed a striking enrichment of CMS4 in the high-risk group, with an Odds Ratio of 4.82. The hypergeometric test confirmed the statistical significance of this result (*p* < 0.0001; Figure 4C).

### 3.4. Superior Prognostic Predictive Performance of CMS-Associated Gene Signature Compared to Oncotype DX and CMS

The clinical value of the CMS-associated gene signature was further validated by comparing it to the FDA-approved Oncotype DX scores [38] and CMSs. Our analysis revealed that our CMS-associated gene signature had the highest mean C-index for prognostic prediction at 0.627, numerically exceeding mean C-indexes of 0.573 and 0.606 for Oncotype DX and CMS, respectively. While we acknowledge that the 95% confidence intervals overlapped in specific cohorts (Figure 4D), suggesting comparable statistical performance in those instances, our gene signature maintained a consistent trend of higher accuracy, achieving the highest C-index level in every cohort except for the Meta-Validation cohort (Table S5). Additionally, to further benchmark the performance, we compared our signature against five other published prognostic signatures for CRC [39,40,41,42,43] and found that our signature numerically outperformed all of them in the training cohort (Figures S4 and S5). In summary, our method of constructing a CMS-associated gene signature significantly differentiated the prognosis of CRC patients and demonstrated robust and competitive performance compared to other methods.

### 3.5. Low-Risk Patients More Likely to Benefit from Immunotherapy

To translate our prognostic findings into clinical management strategies, we evaluated the gene signature’s ability to predict therapeutic responses. Regarding chemotherapy, we screened 48 CRC-associated cell lines and utilized the Genomics of Drug Sensitivity in Cancer (GDSC) database to analyze differences in response to various chemotherapeutic agents between high-risk and low-risk groups. Our results indicated that the low-risk group showcased lower IC50 values for the chemotherapeutic agents Mirin, Gemcitabine, Tamoxifen, and Fludarabine (Figure S6A–D). These findings provide preclinical evidence that low-risk patients might functionally benefit more from these specific regimens, whereas the high-risk group exhibits potential intrinsic resistance, suggesting the need for alternative strategies.

We next investigated whether the low-risk group exhibited a phenotype suitable for immunotherapy. The ssGSEA results showed that the low-risk group had higher CD4 + T cells and CD8 + T cells infiltration levels (Figure S7A), indicating that patients in the low-risk group tend to be “immune hot”.

**Figure 5:**
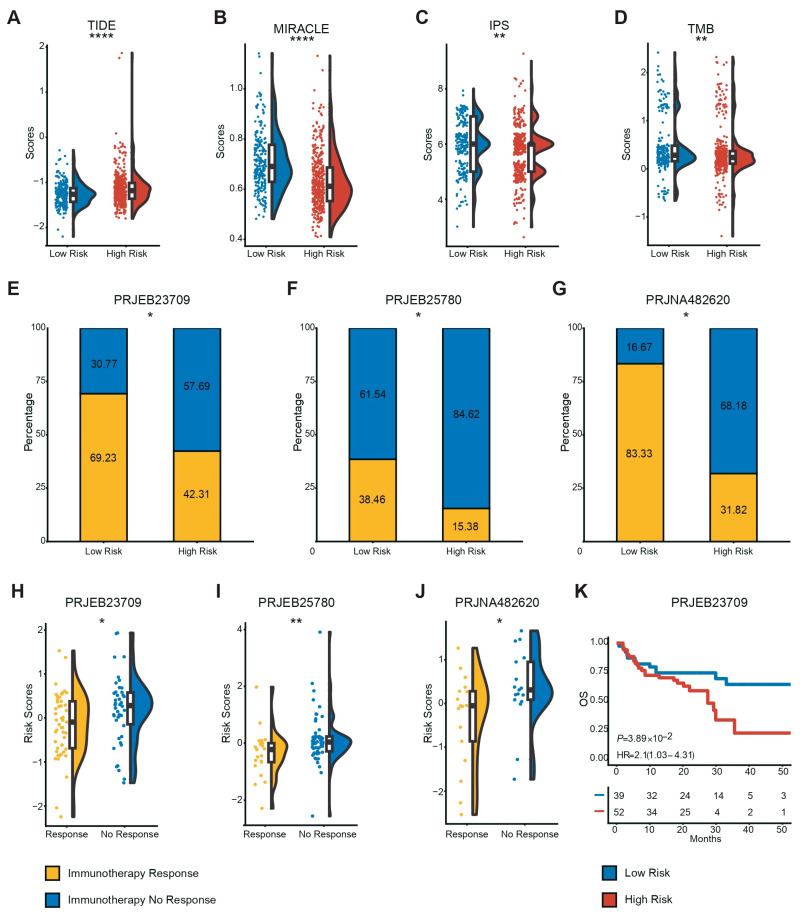
The gene signature predicts favorable therapeutic responses to both chemotherapy and immunotherapy. (**A**–**D**) Distribution of four immunotherapy response scores (TIDE, IPS, MIRACLE, TMB) in different risk groups. (**E**–**J**) Validation in independent immunotherapy cohorts showing objective response rates (**E**–**G**) and risk score distribution (**H**–**J**). (**K**) Kaplan–Meier analysis of the PRJEB23709 cohort. (“*” stands for statistically significant, “*” *p*  <  0.05, “**” *p*  <  0.01, “****” *p*  <  0.0001).

To accurately predict clinical response, we prioritized the Tumor Immune Dysfunction and Exclusion (TIDE) algorithm as the primary benchmark, given its robust predictive value. The analysis demonstrated that the low-risk group had significantly lower TIDE scores (Figure 5A), suggesting a lower potential for immune evasion and a higher likelihood of responding to immunotherapy. To corroborate this finding across multiple dimensions, we further evaluated other established metrics, including IPS, MIRACLE, and TMB. Consistently, these convergent indices aligned with the TIDE results, confirming that patients in the low-risk group have a more favorable immunotherapeutic profile (Figure 5B–D). Further supporting this, we analyzed the expression of immune checkpoints and found that PD-L1, BTLA, LAG3, and TIGIT were more highly expressed in the low-risk group (Figure S7B). These convergent lines of evidence strongly suggest that the low-risk group possesses an inflamed tumor microenvironment favorable for immunotherapy.

To validate these findings in a clinical setting, we utilized the PRJEB23709, PRJEB25780, and PRJNA482620 cohorts to examine the response to immunotherapy among patients predicted to be at low risk or high risk. Notably, a higher proportion of patients in the low-risk group demonstrated a positive response to immunotherapy (Figure 5E–G). More importantly, patients in the immunotherapy-response group exhibited significantly lower risk scores compared to those in the no-response group (Figure 5H–J). In addition, we found that in the PRJEB23709 cohort, which contains survival information, our CMS-associated gene signature also predicted the prognosis of patients well (Figure 5K). Collectively, these findings demonstrate that our signature robustly identifies CRC patients who will derive therapeutic benefit from immunotherapy.

### 3.6. Radiogenomic Signature Predicts Patient Prognosis and Correlates with CMS Pathways

**Figure 6:**
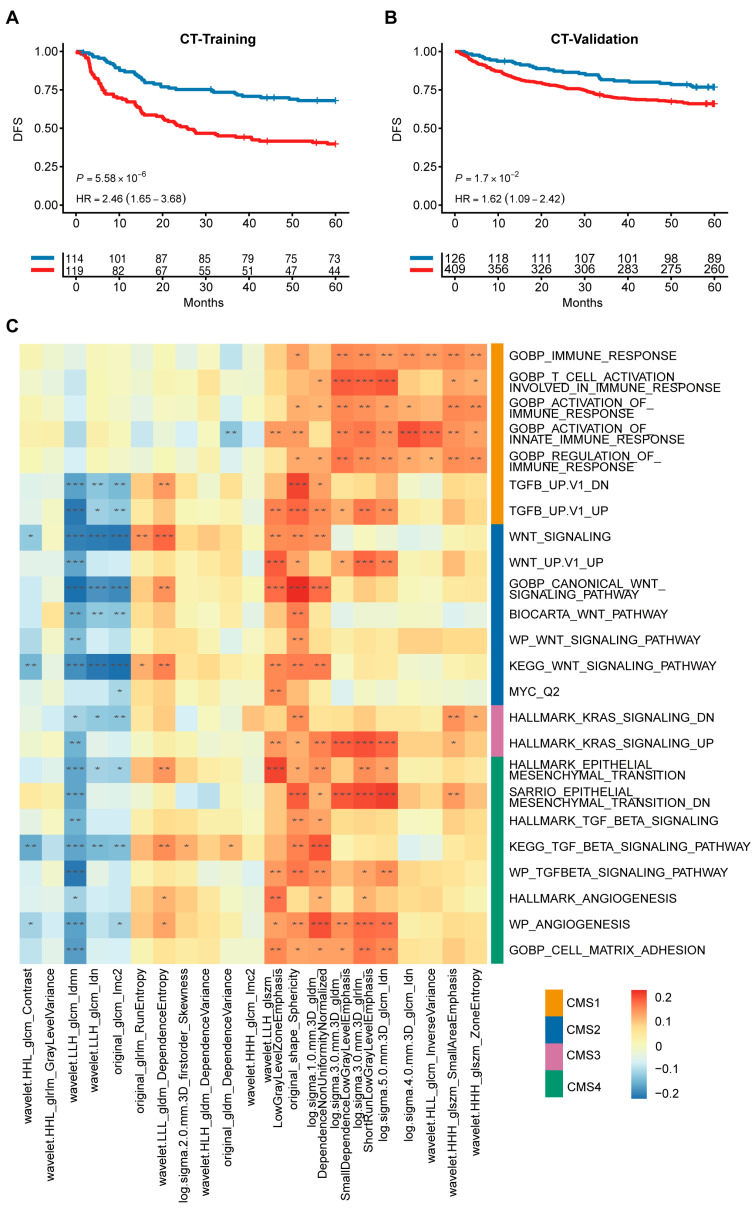
A non-invasive radiogenomic signature predicts prognosis and correlates with molecular pathways. (**A**,**B**) Kaplan–Meier curves show that radiogenomic signature is significantly associated with disease-free survival of patients in the training cohort as well as in the validation cohort. (**C**) Radiogenomic signature significantly correlates with CMS molecular pathways. The color of the color block in the heatmap represents the value of the Pearson correlation coefficient, and ‘*’ represents a statistically significant correlation (“*” *p*  <  0.05, “**” *p*  <  0.01, “***” *p*  <  0.001).

Considering that gene sequencing is more expensive and CT examinations are routinely conducted for CRC patients in clinical practice, we developed a CMS-associated radiogenomic signature using the same pipeline as that used for the gene signature. We correlated the radiomic features extracted with the previously obtained deep features and filtered them to obtain the CMS-associated radiogenomic signature (Table S6). Our results indicated that our radiogenomic signature was also effective in predicting patients’ prognosis (Training Cohort: *p* < 0.0001, HR, 2.46; Validation Cohort: *p* < 0.05, HR, 1.62; Figure 6A,B, Table S7). To directly compare the predictive capability of our two signatures, we evaluated their performance in the 233 patients with matched genomic and radiomic data. While the gene signature demonstrated a higher prognostic accuracy (5-year AUC 0.741), the radiogenomic signature exhibited a moderate reduction in performance (5-year AUC 0.638) yet maintained significant prognostic stratification capabilities. Considering that the radiogenomic signature is non-invasive and derived from routine clinical imaging, it represents a highly valuable and cost-effective alternative for risk assessment in CRC patients (Figure S8).

To interpret the radiogenomic model, we similarly applied SHAP analysis. This identified key radiomic features related to tumor texture, such as wavelet. HHL_glcm_Contrast, as the primary drivers of the risk score (Table S8). Individual patient analyses further demonstrate how these specific imaging characteristics contribute to the final risk stratification, providing transparency to the model’s decision-making process (Figure S9).

To investigate the biological information contained in the radiomic features, we performed a GSEA in patients with matching genomic and radiomic data. RNA-seq data from 233 patients in the training cohort were used to calculate enrichment scores using DeepCC. We selected pathways associated with CMS based on published results from CRCSC and calculated Pearson correlations between 21 radiogenomic features and enrichment scores of specific dysregulated molecular pathways. We found that these 21 radiogenomic features were strongly correlated with CMS-associated molecular pathways. Representatively, Log.sigma.3.0.mm.3D_glrlm_ShortRunLowGrayLevelEmphasis was significantly correlated with immune-related pathways and well represented CMS1, wavelet.LLL_gldm_ DependenceEntropy was highly correlated with the WNT pathway and matched the biology of CMS2, while log.sigma.1.0.mm.3D_gldm_DependenceNonUniformityNormalized correlated with the EMT and TGF-beta pathways and may be associated with CMS4 (Figure 6C). These representative associations provide a robust biological framework for the radiogenomic signature, demonstrating its capacity to reflect the underlying molecular landscape of CRC.

## 4. Discussion

In this large-scale study involving 2948 colorectal cancer patients from 8 cohorts, we introduce a novel framework to translate complex molecular subtypes into clinically practical and cost-effective biomarkers. The core of our work is this transformation framework, which allows for the development of prognostic signatures that accurately predict patient outcomes while addressing a key barrier to clinical implementation by significantly reducing healthcare costs.

The heterogeneity of CRC often results in poor patient survival, necessitating clinically actionable biomarkers. Although Oncotype DX [38] and various other multigene signatures [39,40,41,42,43] have been developed for risk stratification, they often entail high costs or lack direct linkage to the consensus molecular subtypes (CMS). While existing CMS-based prognostic approaches provide deep biological insights, they are often hindered by the high costs and computational complexity of high-throughput sequencing [44]. Our framework offers a more pragmatic alternative. By distilling complex molecular landscapes into a low-throughput, CMS-associated gene signature, we bridge the gap between biological insight and clinical feasibility. Unlike traditional CMS classification, which functions primarily as a research stratification tool, our simplified signature maintains high prognostic fidelity while being compatible with targeted PCR panels. This transition not only significantly reduces the economic burden on healthcare systems but also ensures broader clinical accessibility. The strong correlation between our signature and the original CMS biological categories provides a robust rationale for this streamlined approach, effectively capturing the essential prognostic essence of the CMS framework in a cost-effective format.

Furthermore, our CMS-associated gene signature suggests potential utility in guiding immunotherapy. We observed that patients in the low-risk group were more likely to exhibit favorable immune responses, a finding that aligns with the established biological landscapes of CMS. Specifically, just as CMS1 is characterized by immune infiltration and CMS4 by immune suppression [11,45], our low-risk group was enriched for CMS1, whereas the high-risk group was dominated by CMS4 and showed suboptimal predicted responses. However, we acknowledge that these associations are derived from surrogate efficacy scores in retrospective datasets; thus, they should be interpreted with caution pending prospective validation. Despite this limitation, the results imply that our translational framework effectively captures the underlying biological heterogeneity, bridging the gap between molecular mechanisms and clinical application.

Radiogenomics not only offers valuable insights into gene-based biology but also boasts non-invasive, cost-effective, and convenient advantages. This represents a distinct novelty compared to traditional tissue-based panels, offering a unique opportunity for non-invasive prognostic stratification. Drawing upon this concept, we have employed our translational framework to further convert CRC molecular subtypes into a CMS-associated radiogenomic signature. By doing so, we successfully achieved the goal of accurately predicting the prognosis of CRC patients while minimizing costs and ensuring clinical utility and generalizability. Specifically, this radiogenomic approach incurs virtually no additional financial burden as it utilizes standard-of-care CT scans. Moreover, we identified a strong correlation between radiomic features and biological pathway features, such as WNT, KRAS, Angiogenesis, and Epithelial–Mesenchymal Transition (EMT), which have also been established as biologically characteristic of various CMSs. The integration of genomics and radiomics provides compelling biological interpretability and robust supporting evidence for the clinical application of CMS-associated radiogenomic signatures.

Based on these considerations of cost and accessibility, we propose a practical two-stage workflow to integrate these biomarkers into routine clinical practice. In the preoperative phase, the radiogenomic signature can be derived directly from standard diagnostic CT scans. Since preoperative CT is clinically mandatory, this approach incurs almost no additional cost and offers significant convenience, allowing clinicians to rapidly assess prognostic risk and formulate optimal neoadjuvant strategies. In the postoperative phase, our developed low-throughput gene panel serves as a cost-effective tool for patients with resected tissue. It provides multi-dimensional guidance by not only refining prognostic prediction but also identifying patients likely to benefit from immunotherapy, thereby optimizing adjuvant treatment decision-making.

However, our study has limitations that outline critical directions for future research. First, as a retrospective multi-cohort analysis, potential feature-selection biases and cohort imbalances regarding sample sizes and event rates may exist. Although our signature outperformed comparators, the moderate AUC values observed reflect the intrinsic complexity of CRC prognosis, necessitating validation through prospective clinical trials to definitively confirm utility. Second, regarding radiomics, technical challenges remain; features can be sensitive to image acquisition variations, such as scanner manufacturers and reconstruction kernels. While we applied normalization, future studies must adopt standardized protocols to ensure predictive consistency. Third, the current manual delineation of ROIs is time-consuming, highlighting the need for fully automated AI-based segmentation for clinical scalability. Finally, while we observed strong biological correlations, future studies integrating spatial transcriptomics are needed to further elucidate the precise underlying mechanisms.

## 5. Conclusions

In conclusion, our study demonstrates the feasibility of translating CRC consensus molecular subtypes into cost-effective prognostic biomarkers. Our findings suggest that this framework holds potential for reducing healthcare costs and may offer valuable guidance for diagnostic and therapeutic decisions upon further validation. This work opens up new avenues for cancer biomarker research and could be extended to other cancers for precision treatment.

## Figures and Tables

**Table 1:** Characteristics of patients in all cohorts.

b	TCGA (*n* = 619)	GSE39582 (*n* = 562)	Meta-Validation (*n* = 645)	COCC (*n* = 587)	Training (*n* = 233)	Validation (*n* = 535)	*p*-Value
**Age**							
<65 years old	249 (40.2%)	211 (37.5%)	275 (42.6%)	388 (66.1%)	166 (71.2%)	208 (38.9%)	<0.001
≥65 years old	370 (59.8%)	350 (62.3%)	370 (57.4%)	192 (32.7%)	66 (28.3%)	132 (24.7%)	
**Sex**							
Male	329 (53.2%)	309 (55.0%)	333 (51.6%)	342 (58.3%)	133 (57.1%)	312 (58.3%)	0.107
Female	290 (46.8%)	253 (45.0%)	312 (48.4%)	245 (41.7%)	100 (42.9%)	223 (41.7%)	
**TNM Stage**							
I	105 (17.0%)	32 (5.7%)	69 (10.7%)	67 (11.4%)	22 (9.4%)	82 (15.3%)	<0.001
II	228 (36.8%)	262 (46.6%)	326 (50.5%)	201 (34.2%)	65 (27.9%)	186 (34.8%)	
III	179 (28.9%)	204 (36.3%)	223 (34.6%)	152 (25.9%)	64 (27.5%)	189 (35.3%)	
IV	88 (14.2%)	60 (10.7%)	27 (4.2%)	160 (27.3%)	79 (33.9%)	78 (14.6%)	
**T Stage**							
T1	21 (3.4%)	12 (2.1%)	-	23 (3.9%)	7 (3.0%)	26 (4.9%)	<0.001
T2	105 (17.0%)	44 (7.8%)	-	60 (10.2%)	19 (8.2%)	70 (13.1%)	
T3	422 (68.2%)	364 (64.8%)	82 (12.7%)	400 (68.1%)	167 (71.7%)	373 (69.7%)	
T4	70 (11.3%)	119 (21.2%)	7 (1.1%)	92 (15.7%)	37 (15.9%)	65 (12.1%)	
**N Stage**							
N0	351 (56.7%)	299 (53.2%)	89 (13.8%)	294 (50.1%)	105 (45.1%)	284 (53.1%)	<0.001
N1	150 (24.2%)	133 (23.7%)	-	191 (32.5%)	79 (33.9%)	176 (32.9%)	
N2	115 (18.6%)	98 (17.4%)	-	97 (16.5%)	49 (21.0%)	71 (13.3%)	
**M Stage**							
M0	459 (74.2)	479 (85.2)	89 (13.8)	422 (71.9)	154 (66.1)	457 (85.4%)	<0.001
M1	87 (14.1)	61 (10.9)	0 (0.0)	118 (20.1)	59 (25.3)	77 (14.4%)	
**MSS/MSI Status**							
MSI	188 (30.4%)	72 (12.8%)	33 (5.1%)	53 (9.0%)	13 (5.6%)	85 (15.9%)	<0.001
MSS	428 (69.1%)	444 (79.0%)	95 (14.7%)	496 (84.5%)	207 (88.8%)	264 (49.3%)	
**CMS Stage**							
CMS1	68 (11.0%)	89 (15.8%)	130 (20.2%)	73 (12.4%)	16 (6.9%)	-	<0.001
CMS2	206 (33.3%)	232 (41.3%)	237 (36.7%)	233 (39.7%)	73 (31.3%)	-	
CMS3	64 (10.3%)	69 (12.3%)	100 (15.5%)	93 (15.8%)	26 (11.2%)	-	
CMS4	116 (18.7%)	126 (22.4%)	134 (20.8%)	180 (30.7%)	69 (29.6%)	-	
**Tumor Location**							
Right	270 (43.6%)	220 (39.1%)	160 (24.8%)	134 (22.8%)	-	-	<0.001
Left	349 (56.4%)	342 (60.9%)	195 (30.2%)	410 (69.8%)	-	-	

## Data Availability

The data generated from this study are publicly available in the Firehose Broad GDAC portal and in Gene Expression Omnibus (GEO) at GSE39582, GSE14333, GSE17538, GSE33113, GSE37892, GSE38832, GSE31595, GSE75316. COCC RNA-seq data and CT scans data from the Sixth Affiliated Hospital of Sun Yat-sen University are not publicly available but can be obtained from the corresponding author on reasonable request.

## Supplementary Materials

The following supporting information can be downloaded at https://www.mdpi.com/article/10.3390/diagnostics16020273/s1, Figure S1: Validation of the gene signature in the four individual Meta-Validation cohorts; Figure S2: Validation of the gene signature in three additional independent cohorts; Figure S3: Interpretability of the CMS-associated gene signature using SHAP analysis; Figure S4: Prognostic performance of three other published signatures (Mo-Ol, Dekervel, Lin-JTM) evaluated in the TCGA training cohort; Figure S5: Prognostic performance of two other published signatures (Hao, Gharib) evaluated in the TCGA training cohort; Figure S6: Drug sensitivity analysis in CRC cell lines; Figure S7: Immune cell infiltration and expression of immune checkpoints; Figure S8: Comparative prognostic performance of the radiogenomic signature and the gene signature in the matched CT-Training cohort; Figure S9: Interpretability of the CMS-associated radiogenomic signature using SHAP analysis; Table S1: CMS-Associated Gene Signature; Table S2: Factor Analysis for CMS-Associated Gene Signature; Table S3: Genomics Signature SHAP Feature Importance; Table S4: Literature-based validation for the biological roles of impactful genes identified by SHAP analysis; Table S5: C-index for Prognosis Prediction; Table S6: CMS-Associated Radiogenomic Signature; Table S7: Factor Analysis for CMS-Associated Radiogenomic Signature; Table S8: Radiogenomics Signature SHAP Feature Importance.

## Abbreviations

The following abbreviations are used in this manuscript:

CRC	Colorectal cancer
CRCSC	Colorectal Cancer Subtyping Consortium
CMS	Consensus molecular subtypes
GEO	Gene Expression Omnibus
COCC	Clinical Genomic Study of Colorectal Cancer in China
ICGC-ARGO	International Cancer Genome Consortium to Accelerate Research in Genomic Oncology
DICOM	Digital Imaging and Communication in Medicine
GSEA	Gene set enrichment analysis
ssGSEA	single-sample gene set enrichment analysis
TIGER	Tumor Immunotherapy Gene Expression Resource
UMAP	Uniform manifold approximation and projection
RS	Risk score
DFS	Disease-free survival
EMT	Epithelial–mesenchymal transition
