# Supplementary material for: Translating Molecular Subtypes into Cost-Effective Radiogenomic Biomarkers for Prognosis of Colorectal Cancer
Source: Diagnostics (Basel). 2026 Jan 14;16(2):273. doi: 10.3390/diagnostics16020273 (PMC12839849; doi:10.3390/diagnostics16020273)
Supplement: Supplementary file 1 [file diagnostics-16-00273-s001.zip › Supplementary Figure.pdf]

Supplementary Figure

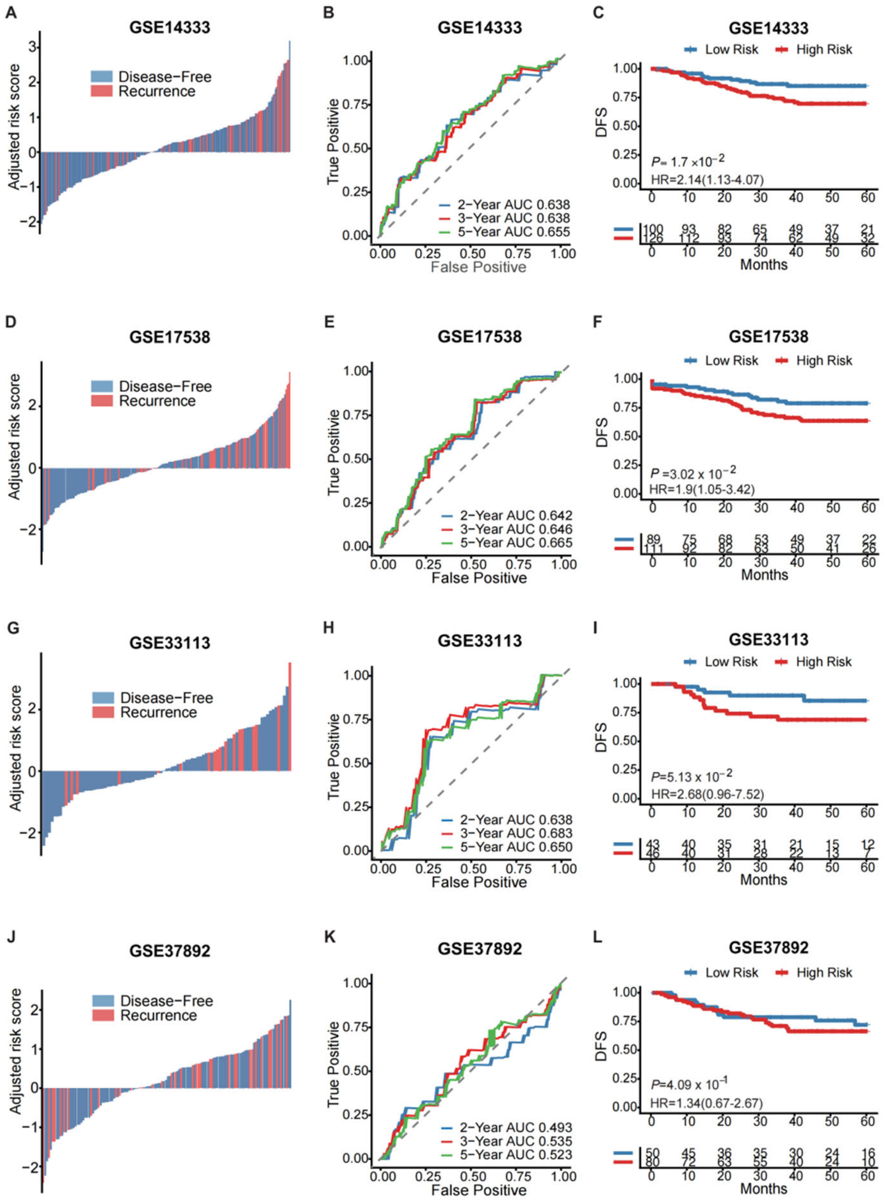

Fig S1. Validation of the gene signature in the four individual Meta-Validation cohorts. **(A, D, G, J)** Waterfall plots show the relationship between gene signature risk scores and recurrence status in the GSE14333, GSE17538, GSE33113, and GSE37892 cohorts, respectively. **(B, E, H, K)** Time-dependent ROC curves show the ability of the gene signature to discriminate recurrence status at 2, 3, and 5 years. **(C, F, I, L)** Kaplan-Meier curves reveal associations between the gene signature and disease-free survival (DFS) in all four cohorts

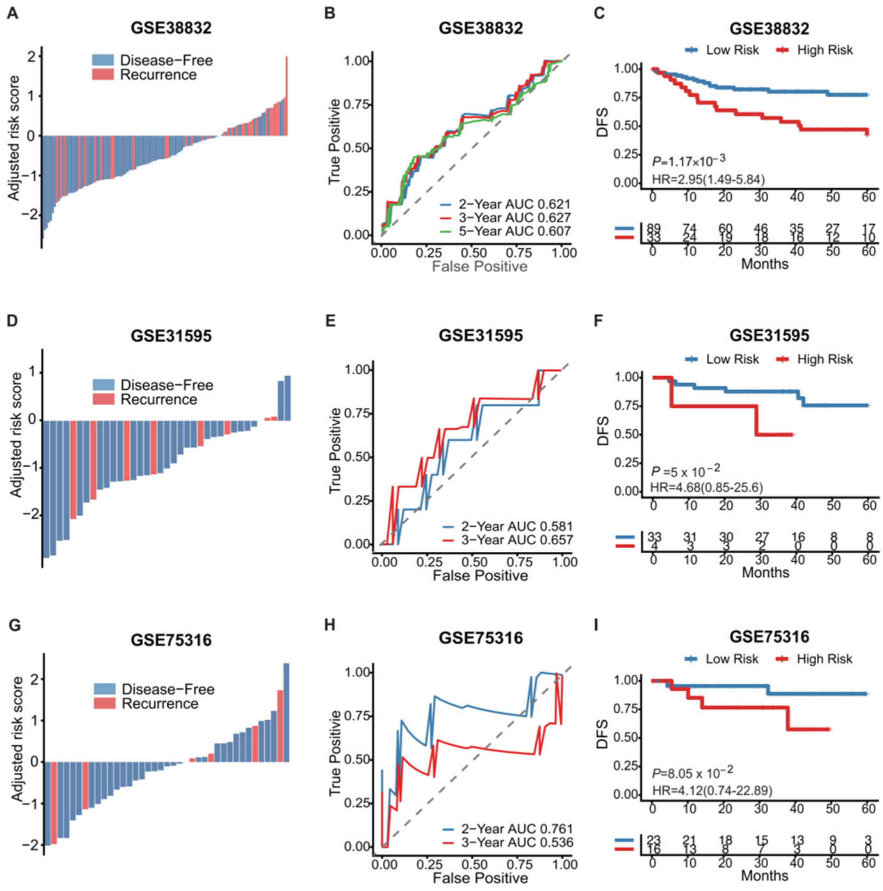

Fig S2. Validation of the gene signature in three additional independent cohorts. **(A, D, G)** Waterfall plots show the relationship between gene signature risk scores and recurrence status in the GSE38832, GSE31595, and GSE75316 cohorts, respectively. **(B, E, H)** Time-dependent ROC curves show the ability of the gene signature to discriminate recurrence status. **(C, F, I)** Kaplan-Meier curves for disease-free survival (DFS) based on the signature-defined high- and low-risk groups.

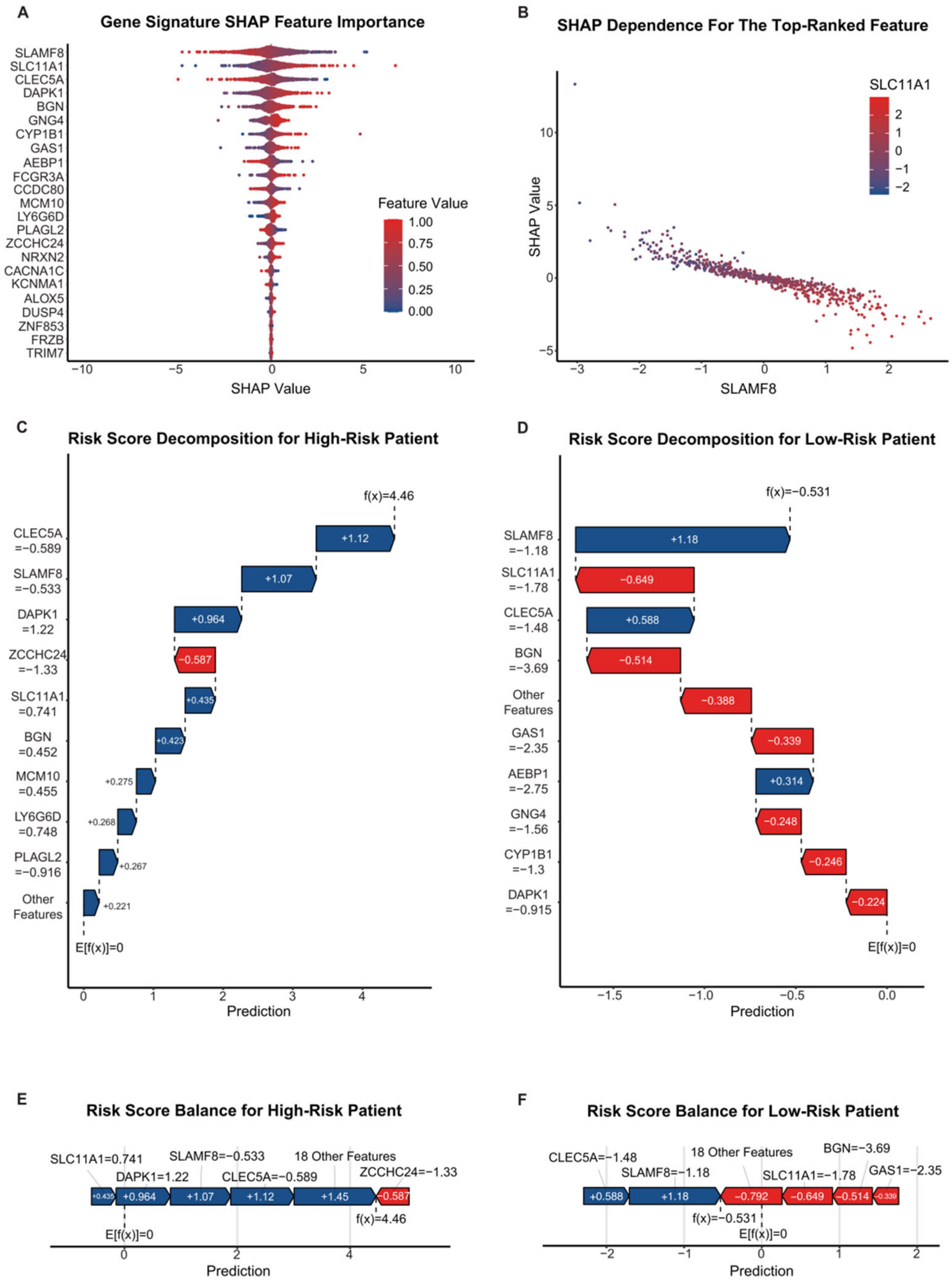

Fig S3. Interpretability of the CMS-associated gene signature using SHAP analysis. **(A)** SHAP summary plot illustrating the overall impact of each gene in the signature. Each dot represents a patient, with color indicating the feature value (red=high expression, blue=low). **(B)** SHAP dependence plot for the top-ranked feature (SLAMF8), showing its interaction with SLC11A1. **(C, E)** Risk decomposition and risk balance for a representative high-risk patient, showing how each gene's expression contributes to the final prediction. **(D, F)** Risk decomposition and risk balance for a representative low-risk patient.

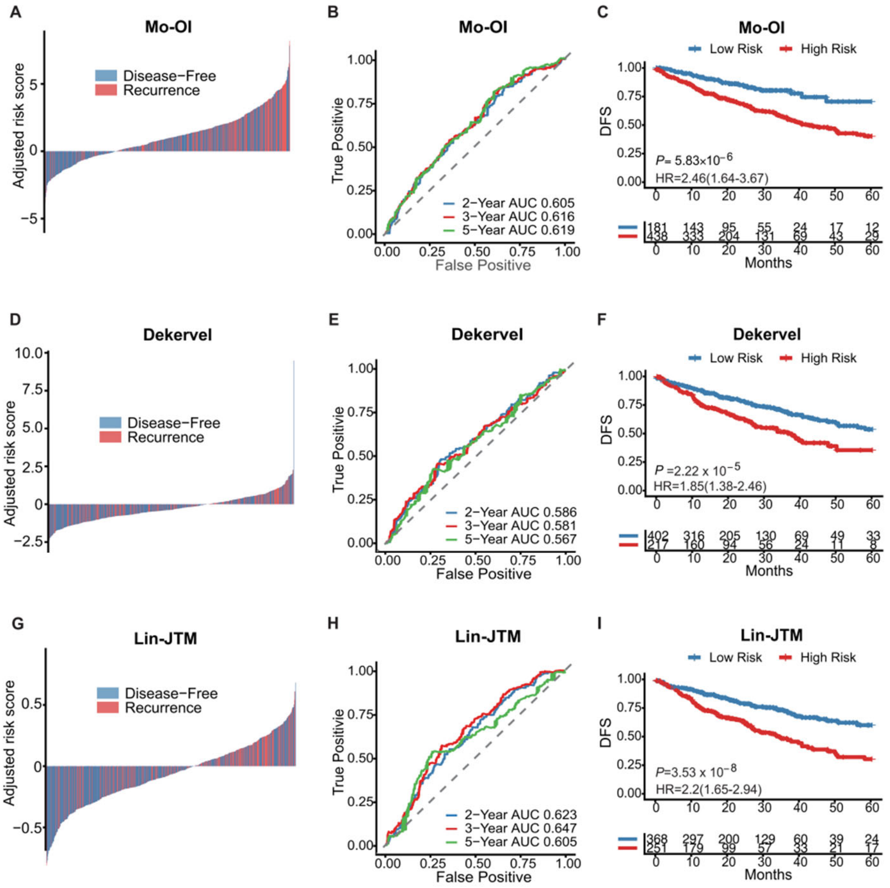

Fig S4. Prognostic performance of three other published signatures (Mo-Ol, Dekervel, Lin-JTM) evaluated in the TCGA training cohort. **(A, D, G)** Waterfall plots showing the risk score distribution and recurrence status for the Mo-Ol, Dekervel, and Lin-JTM signatures. **(B, E, H)** Time-dependent ROC curves for the three signatures. **(C, F, I)** Kaplan-Meier curves for disease-free survival (DFS) stratified by the three signatures.

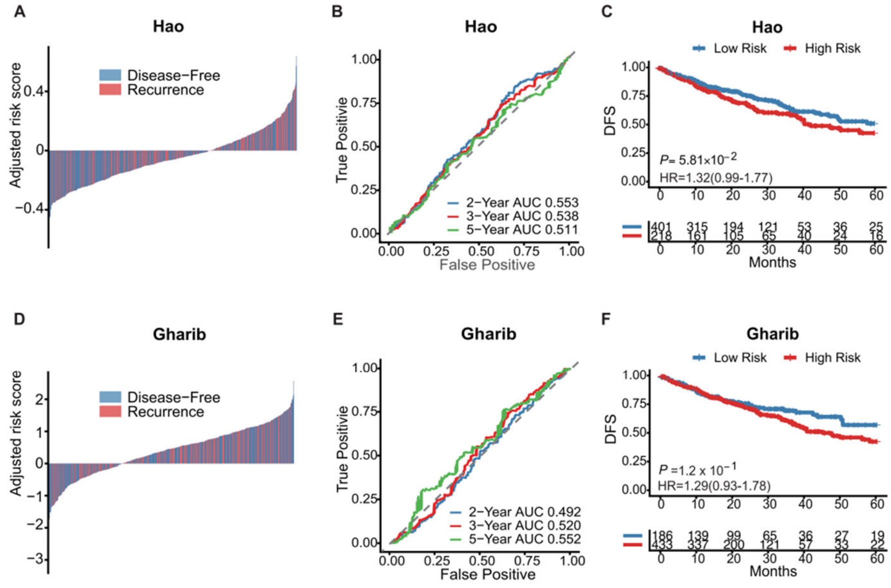

Fig S5. Prognostic performance of two other published signatures (Hao, Gharib) evaluated in the TCGA training cohort. **(A, D)** Waterfall plots for the Hao and Gharib signatures. **(B, E)** Time-dependent ROC curves for the two signatures. **(C, F)** Kaplan-Meier curves for disease-free survival (DFS) stratified by the two signatures.

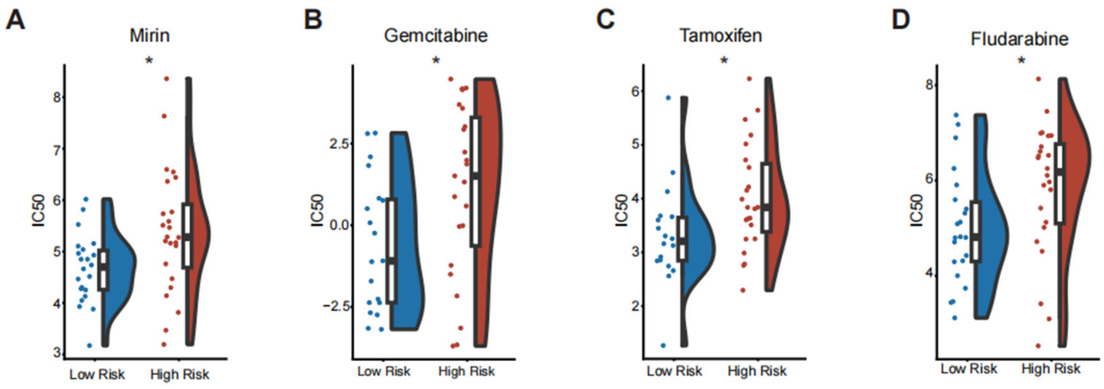

Figure S6. Drug sensitivity analysis in CRC cell lines. **(A, B, C, D)** Violin plots showing the IC50 values of Mirin, Gemcitabine, Tamoxifen, and Fludarabine in high- and low-risk groups. The high-risk group exhibits significantly higher IC50 values, indicating potential resistance. (*P < 0.05).

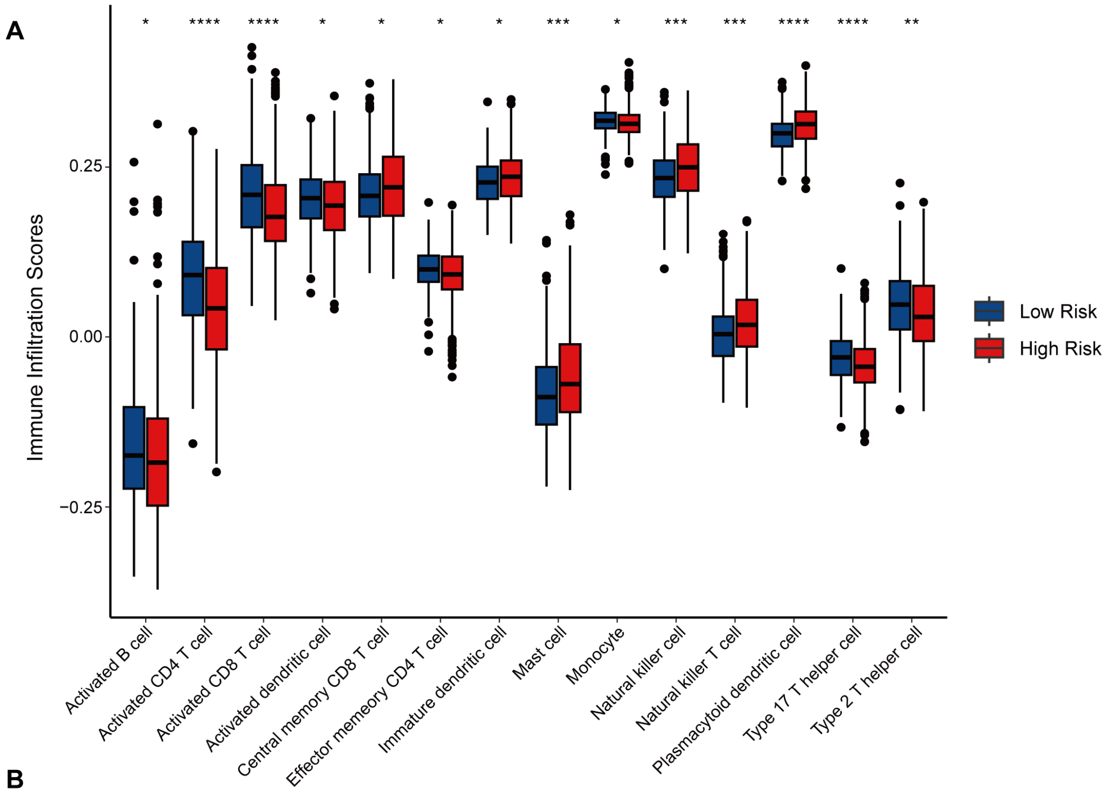

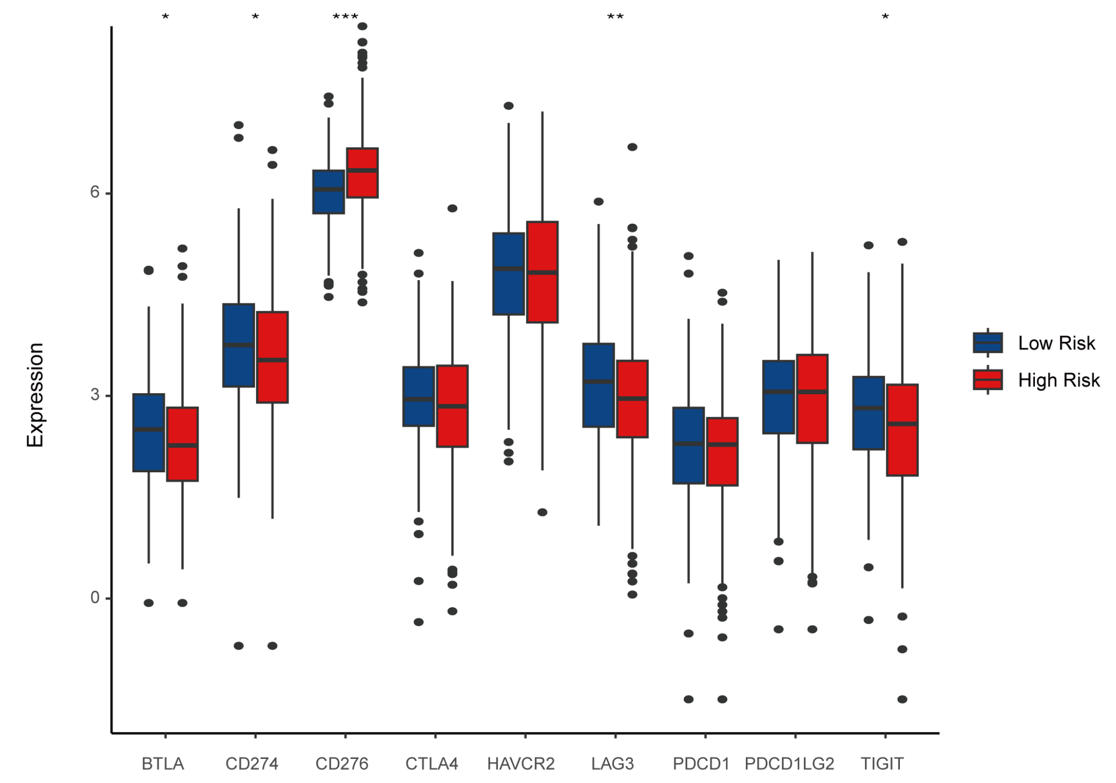

Fig S7. Immune cell infiltration and expression of immune checkpoints. **(A)** Immune cell infiltration scores between high and low risk groups. **(B)** Immune checkpoint gene expression between high and low risk groups. ("*" stands for statistically significant, “*” P < 0.05, “**” P < 0.01, “***” P < 0.001, “****” P < 0.0001)

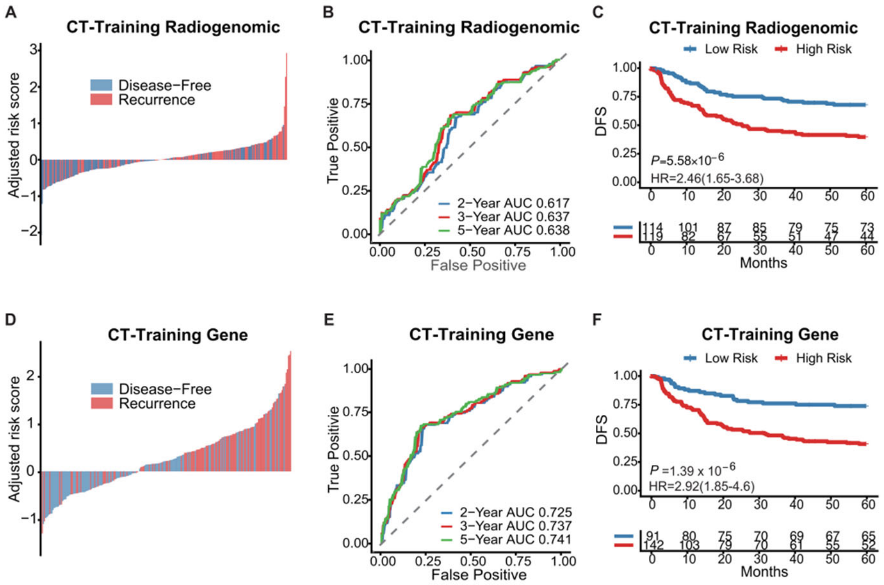

Fig S8. Comparative prognostic performance of the radiogenomic signature and the gene signature in the matched CT-Training cohort. **(A, B, C)** Waterfall plot, time-dependent ROC curves, and Kaplan-Meier curve for the radiogenomic signature. **(D, E, F)** Waterfall plot, time-dependent ROC curves, and Kaplan-Meier curve for the gene signature in the same patient cohort, allowing for direct comparison.

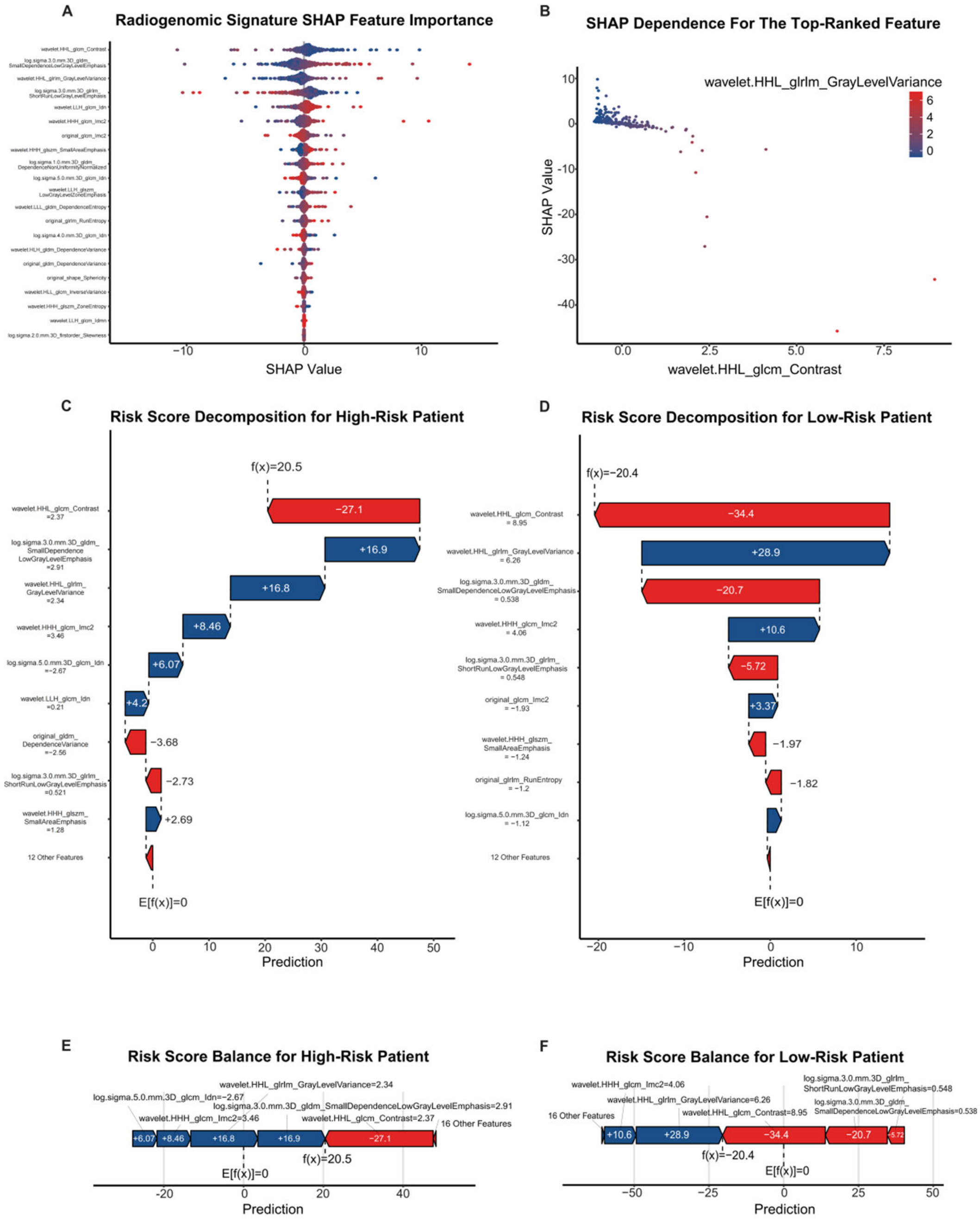

Fig S9. Interpretability of the CMS-associated radiogenomic signature using SHAP analysis. **(A)** SHAP summary plot illustrating the overall impact of each radiomic feature on the model's output. **(B)** SHAP dependence plot for the top-ranked radiomic feature. **(C, E)** Risk decomposition and risk balance for a representative high-risk patient. **(D, F)** Risk decomposition and risk balance for a representative low-risk patient.
